# New Records of Lake Baikal Leech Fauna: Species Diversity and Spatial Distribution in Chivyrkuy Gulf

**DOI:** 10.1155/2013/206590

**Published:** 2013-06-06

**Authors:** Irina A. Kaygorodova, Nikolay M. Pronin

**Affiliations:** ^1^Limnological Institute of Siberian Branch of Russian Academy of Sciences, 3 Ulan-Batorskaja Street, Irkutsk 664033, Russia; ^2^Institute of General and Experimental Biology of Siberian Branch of Russian Academy of Sciences, 6 Sakhyanova Street, Ulan-Ude 670047, Russia

## Abstract

The study of several Lake Baikal leech collections offered us the possibility to determine species diversity in the Chivyrkuy Gulf, the biggest one in the lake. As a result, the first information on the Chivyrkuy Hirudinea fauna (Annelida, Clitellata) has been revealed. There are two orders and four families of leeches in the Chivyrkuy Gulf: order Rhynchobdellida (families Glossiphoniidae and Piscicolidae) and order Arhynchobdellida (families Erpobdellidae and Haemopidae). In total, 22 leech species and 2 subspecies belonging to 11 genera were identified. Of these, 4 taxa belong to the family Glossiphoniidae (*G. concolor*, *A. hyalina*, *A. heteroclita* f. *papillosa*, and *A. heteroclita* f. *striata*) recorded in Baikal for the first time. Representatives of 8 unidentified species (*Glossophinia* sp., *Baicaloclepsis* sp., *Baicalobdella* sp., *Piscicola* sp. 1, *Piscicola* sp. 2, *Erpobdella* sp. 1, *Erpobdella* sp. 2, and *Erpobdella* sp. 3) have been also recorded. The checklist gives a contemporary overview of the species composition of leech parasites, their hosts, and distribution within the Chivyrkuy Gulf. The analysis of spatial distribution has shown that the leech species diversity is correlated with the biological productivity of the bay. The most diverse community of leech species is detected in the eutrophic zone of the lake.

## 1. Introduction

The intensive 200-year study of Lake Baikal found that it is the oldest (25–30 million years), the deepest (1637 m), and the largest (23 000 cubic km) repository of fresh water on the planet (20% affordable drinking water), with the status of the Natural World Heritage sites (Merida, 1996). Although Baikal is one of the most studied lakes in the world, there are still “white” spots. One of them is the Chivyrkuy Gulf, the largest and the least studied part of the lake. Nevertheless, the Chivyrkuy Gulf is a unique ecosystem. Not by chance, the prominent limnologist and Baikal researcher Vereshchagin, relying on the results of his first Circum-Baikal expedition, came to an unambiguous conclusion that the best place to study activities at Lake Baikal was the Holy Nose Peninsula, which forms the western shore of the Chivyrkuy Gulf [1]. However, in those days, the proximity of transport communications played a decisive role in establishing scientific stations in the southern Baikal (Bolshie Koty, Listvyanka). Therefore, the Chivyrkuy Gulf stood aside from the routes of the majority of academic expeditions for a long time.

**Figure 1:**
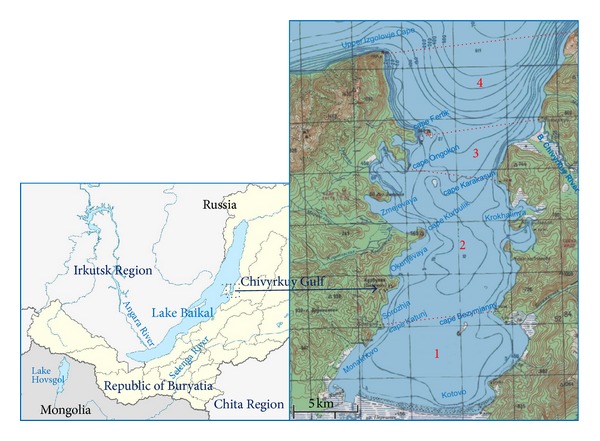
Geographical location of the study region with bathymetric data and indication of main bays and capes. Dotted lines divide different bioproductivity zones: 1: eutrophic zone, 2: mesotrophic zone, 3: oligotrophic zone, and 4: ultraoligotrophic zone.

The Chivyrkuy Gulf is located in the north-eastern part of Lake Baikal (Figure 1). With an area of about 270 km^2^, maximal width of 13 km, and length of 27 km, the Chivyrkuy Gulf is the most deeply intrusive and isolated gulf in Lake Baikal [1]. The western and eastern shores of the gulf form a chain of bays (Figure 1). Chivyrkuy Gulf has a uniform bottom rising deep into the gulf. Only in its transit there are depths of over 100 m. Most of the gulf has a depth of less than 25 m [2]. In its low-lying water-logged part of the south shore (with a depth of up to 4 m), there is a runoff from the adjacent Lake Arangatuy. The bottom of Chivyrkuy is predominantly sandy and, only at the outlet of the gulf, at a depth of more than 20–30 m is covered with silted sand and then grey silt [3]. Black or brown silt covers the bottom of the multiple bays. Rocky bottoms are poorly represented.

Some features of morphometry, temperature, and glacial regimes, and certain hydrobiological characteristics allow the Chivyrkuy Gulf to be regarded as an ecosystem with a natural succession of zones of different biological productivity. The transect “Lake Arangatuy-Chivyrkuy Bay-Baikal proper” demonstrates a full range of conditions for transition from the open waters of Lake Baikal to the coastal sor zone (in the Russian scientific literature, sors are called closed, small, usually well-warmed bays of Lake Baikal) with the change of the bioproductivity type of waters and biota (Figure 1).

Ecological and geographic features of the Chivyrkuy Gulf, such as the shallow depth, high resistance to winds, rich benthic vegetation, high oxygen content, good water warming, and significant amounts of detritus in the substrate, create favourable environmental conditions for development of zoobenthic fauna and fish. In spite of abundant and variable biological resources, few papers have been devoted to fauna of the Chivyrkuy Gulf [1, 4–7]. Later these materials were summarized and included in the Kozhov's monograph [2]. Further biological studies of the Chivyrkuy Gulf were mainly restricted to certain taxonomic groups. Data on species composition, abundance, and distribution of Amphipoda [8], Flagellata [9], Bivalvia [10], and Oligochaeta [11] were obtained.

The parasite fauna of Lake Baikal consists of 280 species [12]. Baikal leeches have never been the subject of special study. Hence, there are conflicting data on the fauna composition even in a single publication [13]. The study of fauna and ecology of Baikal leeches is topical as they are the definitive hosts of parasitic flagellates of Trypanosomatida and Bodomonadida [14, 15]. The latest studies of the Hirudinea fauna demonstrate taxonomical diversity of leeches in the Baikal [16, 17].

This paper presents the first data on leech species diversity and its spatial distribution within the Chivyrkuy Gulf and, on the other hand, continues the series of publications aiming at updating knowledge on Lake Baikal fauna.

## 2. Material and Methods

Over a 15-year period, leeches were collected using every possible means during hydrobiologic and ichthyologic studies by Dr. Nikolay Pronin (1996–2012) and during targeted sampling of leeches by Dr. Irina Kaygorodova (2011-2012). Field works were carried out at the biological station in the Chivyrkuy Gulf which belongs to the Institute of General and Experimental Biology.

It is well known that leeches have suckers and can spend much of their life in the attached state. Therefore, usual gears (sweep net, dredge, scraper, bottom grab, etc.) are often less effective in procuring leeches than searching for many other aquatic invertebrates. To catch parasitic and predatory leech species we inspected various aquatic plants and animals as well as different underwater objects (rotten wood, driftwood, snags, stones, etc.), to which hirudinids can be attached. Some leeches were picked out from qualitative and quantitative zoobenthic samples. In most cases piscine leeches were gathered directly in captured living fish. Fish were caught using different fishing tackles such as a fishing net and a fishing rod. Additionally, professional scuba divers helped in leech sampling at a depth of 3–50 m.

All specimens were placed in separate vials and kept in either 4% formaldehyde solution (old samples) or 80% ethanol solution. Each of these preserving methods has its own advantages and disadvantages. Formalin preserves body colour and eyes better and condenses elements of the reproductive system, which makes dissection and morphological analysis easier. However, formalin-fixed leeches have often a strong contraction of the body making it difficult to visualize genital pores, the location of which is an important feature for determining systematic position. Alcoholised leech tissues retain flexibility, making investigation of annulation and allocation of genital openings easier. Moreover, such material is suitable for further molecular analysis.

Morphological analysis was performed using a stereomicroscope MSP-2 var. 2 (LOMO). Currently existent systematic keys [18–20] and original taxonomical descriptions [21, 22] were used for parasite species identification. Many specimens were not identified to the species level, even in groups in which this is theoretically possible. Publication of this list could be delayed for long to await more comprehensive accuracy; however, enough significant data had already been accumulated to be published.

The examined material is deposited in the Limnological Institute, Siberian Branch of the Russian Academy of Sciences (LIN SB RAS).

## 3. Results

Extensive collection of parasitic annelids (Hirudinea, Clitellata) and their hosts has been going for many years of research in the Chivyrkuy Gulf. Morphological analysis of this collection has been completed recently. The results are presented as a checklist of leech species inhabiting the Chivyrkuy Gulf of Lake Baikal. The list was composed of 22 species and 2 subspecies. The exact systematic position of each taxon and its taxonomic hierarchy data are given in Table 1.

### 3.1. Taxonomic Review and a Brief Description of Each Taxon

In a brief commentary, we include a concise description of each leech species with emphasis on host-parasite relationship, zoogeographical and ecological characteristics, the occurrence of species, and its spatial distribution within the Chivyrkuy Gulf. Numerical evaluation of biodiversity could be useful for understanding the importance of species number in terms of actual biodiversity of parasites. The classification of fish taxa is given according to Bogutskaya and Naseka [23].

#### 3.1.1. *Theromyzon maculosum* (Rathke, 1862)

 Local host: unknown.
 Locality: Monakhovo Bay (NGR).

A widespread but rarely found Palaearctic species. It is known as the bloodsucker of swimming birds inhabiting warm shallow gulfs of Lake Baikal [18, 24]. Two individuals of this species were found in the coastal part of transect 1 (Figure 1) at a depth of 1–3 m during zoobenthic study. The body size of collected specimens was 20–25 mm.

#### 3.1.2. *Theromyzon tessulatum* (Müller, 1774)

 Local host: unknown.
 Locality: Chivyrkuy Gulf (?).

This Palaearctic species is known as the bloodsucker of waterfowl [24]. It was not recorded in samples from the Chivyrkuy Gulf. Nevertheless, there are all suitable environmental conditions for existence of *T. tessulatum* in the Chivyrkuy Gulf.

#### 3.1.3. *Hemiclepsis marginata* (Müller, 1774)

 Local host: young fish, invertebrates (NHR).
 Locality: Kotovo Bay (NGR), Zmejovaya Bay (NGR).

A common Palaearctic species. Bloodsucker of fish, tadpoles, and amphipods. Representatives of this species were found at transect 1 (Figure 1) in washout from aquatic vegetation and at transect 2 (Figure 1) on stones at a depth of 0.3–0.7 m. Living leeches are green with a length of 14–16 mm and 3 mm in width. Alcohol fixed specimens rapidly lose their beautiful intravital colouring.

#### 3.1.4. *Helobdella nuda* (Moore, 1924)

 Local host: Mollusca: Gastropoda.
 Locality: Zmejovaya Bay (NGR).

A Palaearctic species inhabiting the Amur River basin. It has been recorded recently in Lake Baikal [25]. We found *H. nuda* at the western point of the Zmejovaya Bay (Figure 1: transect 2). Two individuals were found on the bottom surface of rocks at a depth of 0.7 m. Our new record of *H. nuda* suggests that the range of this species is much wider than it has been considered before and is not restricted to the Sino-Japanese region.

#### 3.1.5. *Helobdella stagnalis* (Linnaeus, 1758)

 Local host: small invertebrates (oligochaetes, larvae of amphibiotic insects, molluscs, young isopods, and amphipods).
 Locality: Kotovo Bay, Monakhovo Bay, Sorozhiya Bay, Okunevaya Bay, Cape Kurbulik, and Zmejovaya Bay.

This species is considered as one of the most common freshwater leeches in the world, a cosmopolite. Within Lake Baikal the *H. stagnalis* inhabits shallow bays and coastal sors. In our collection of the Chivyrkuy Gulf, there are samples only from locations with eutrophic and mesotrophic conditions of the east side of the gulf (Figure 1: transects 1 and 2).

#### 3.1.6. *Glossiphonia complanata* (Linnaeus, 1758)

 Local host: Mollusca: Gastropoda.
 Locality: Monakhovo Bay, Sorozhiya Bay, Okunevaya Bay, Cape Kurbulik, Zmejovaya Bay, and the Krokhalinaya Bay.

A Holarctic species is widespread in Siberia [18]. In exceptional cases, *G. complanata* can parasitize annelid worms, larvae of amphibiotic insects, fish eggs, and cocoons of leeches [26]. Specimens were caught in the coastal zone of the Chivyrkuy Gulf in locations corresponding to eutrophic and mesotrophic water bodies.

#### 3.1.7. *Glossiphonia concolor* (Apáthy, 1888)

 Local host: Mollusca: Gastropoda.
 Locality: Kotovo Bay (NGR), Zmejovaya Bay (NGR).

A Palaearctic species. For the first time found in Baikal. Mature individuals of this species were collected in transects 1 and 2 (Figure 1) in a depth of 0–1.0 m. These leeches prefer sand, pebbles, and flat stones.

#### 3.1.8. *Glossiphonia sp.*

 Local host: Mollusca: Gastropoda (NHPR).
 Locality: Monakhovo Bay, Zmejovaya Bay.

For the first time reported in Baikal. These leeches were found in transects 1 and 2 (Figure 1) in a tangle of elodea and fouling of underwater objects (stones, rotten wood, etc.) in a depth of 0–0.5 m. Representatives of this group have three pairs of eyes with typical location for the genus and atypical dislocation of papillae on the dorsal part of the body. Length and width of the body are 6-7 and 3–5 mm, correspondingly.

#### 3.1.9. *Alboglossiphonia hyalina* (Müller, 1774)

 Local host: Gastropoda, Isopoda, Oligochaeta.
 Locality: Monakhovo Bay (NGR), Cape Kurbulik (NGR), Zmejovaya Bay (NGR), and Krokhalinaya Bay (NGR).

A Holarctic species. Its distribution over a vast area is irregular [18]. *A. hyalina* is a suctorial freshwater sit-and-wait predator, feeding mainly on Gastropoda, Isopoda, and Oligochaeta. It inhabits shallow waters of eutrophic and mesotrophic parts of the Chivyrkuy. In our collection, there are specimens from transects 1 and 2 (Figure 1).

#### 3.1.10. *Alboglossiphonia heteroclita* (Linneaus, 1761)

A widespread Holarctic species. This benthic species preys on small invertebrates. There are two forms of this species, which differ in the amount of pigmentation on the dorsal side of the body.

*A. heteroclita f. papillosa (Braun, 1805)*

 Local host: Mollusca: Gastropoda.
 Locality: Kotovo Bay (NGR), Monakhovo Bay (NGR), Okunevaya Bay (NGR), and Krokhalinaya Bay (NGR).

For the first time listed for Baikal. There is a median row of dark spots. This taxon is recorded in the eutrophic (Figure 1: transect 1) and mesotrophic parts (Figure 1: transect 2) of the Chivyrkuy Gulf.

*A. heteroclita f. striata (Apáthy, 1888)*

 Local host: Mollusca: Gastropoda.
 Locality: Kotovo Bay (NGR), Monakhovo Bay (NGR).

For the first time listed for Baikal. This taxon has elongated stripes in transverse to the body rim [18, 20]. These leeches were found only in the eutrophic part of the Chivyrkuy Gulf (Figure 1: transect 1).

#### 3.1.11. *Baicaloclepsis echinulata* (Grube, 1871)

 Local host: Mollusca: Gastropoda: Benedictiidae (NHR).
 Locality: mouth of the Chivyrkuy Gulf (NGR).

This endemic to Lake Baikal species inhabits the open waters of the lake up to 300 m. Feeding details were unknown. We found them attached on molluscs of genus *Benedictia* in the outlet of the Chivyrkuy Gulf with trawling at a depth of 40–65 m (Figure 1: transect 4).

#### 3.1.12. *Baicaloclepsis sp.*

 Local host: Mollusca: Gastropoda (NHPR).
 Locality: Chivyrkuy Gulf.

For the first time listed for Baikal. The taxon belongs to an endemic genus. One individual was found by dredging in the middle part of transect 3 at a depth of 15.9 m (Figure 1). Morphologically this leech is similar to *B. echinulata* except for small papillae on the ventral side of the body.

#### 3.1.13. *Baicalobdella torquata* (Grube, 1871)

 Local host: Crustacea: Amphipoda.
 Locality: outlet of Chivyrkuy Gulf (NGR).

An endemic species to Lake Baikal. It is a typical component of the littoral zone in the open Baikal. Within the Chivyrkuy Gulf, we found this species only in the oligotrophic part at a depth of 20–65 m (Figure 1: transect 3). *B. torquata* sucks the blood of Baikal endemic amphipods.

#### 3.1.14. *Baicalobdella cottidarum *(Dogiel et Bogolepova, 1957)

 Local host: Scorpaeniformes: Cottidae: Abyssocottinae: *Procottus jeittelesii* (Dybowski, 1874).
 Locality: outlet of Chivyrkuy Gulf (NGR).

An endemic species to Lake Baikal. For the first time listed for the Chivyrkuy Gulf. This species inhabits the northern oligotrophic part of the Chivyrkuy Gulf (Figure 1: transect 3). We caught them at 35 m parasitizing cottoid fish.

#### 3.1.15. *Baicalobdella sp.*

 Local host: Scorpaeniformes: Cottidae: Abyssocottinae (NHPR).
 Locality: outlet of the Chivyrkuy Gulf.

For the first time listed for Baikal. A leech belongs to the Baikal endemic genus. In contrast to *B. torquata*, this species is larger and lacks a characteristic white clitellum. This species inhabits the northern oligotrophic part of the Chivyrkuy Gulf (Figure 1: transect 3). We caught them at 20 m parasitizing cottoid fishes.

#### 3.1.16. *Codonobdella truncata* (Grube, 1872)

 Local host: Crustacea: Amphipoda.
 Locality: Chivyrkuy Gulf (NGR).

An endemic species to Lake Baikal. This species inhabits the abyssal zone of the lake. We found it on deep-water amphipods, which were caught in front of the mouth of the Bolshoi Chivyrkuy River (Figure 1: transect 4) at a depth of 240 m.

#### 3.1.17. *Piscicola sp. 1*

 Local host: Cypriniformes: Cyprinidae: *Rutilus lacustris* (Linnaeus, 1758) (NHPR).
 Locality: Sorozhiya Bay.

Recently reported in Baikal [16]. This is a small-sized leech (length up to 8 mm) having special body coloration different from the widespread species *Piscicola geometra*. Within the Chivyrkuy Gulf, one specimen was found on a roach, which was caught in waters opposite to Cape Kanin (Figure 1: transect 1).

#### 3.1.18. *Piscicola sp. 2*

 Local host: Perciformes: Percidae: *Perca fluviatilis* Linnaeus, 1758 (NHPR).
 Locality: Monakhovo Bay.

First time referred to Baikal. A few specimens were recently found on a perch in transect 1 (Figure 1). Body length is up to 20 mm. This sample requires further study and description.

#### 3.1.19. *Haemopis sanguisuga* (Linnaeus, 1758)

 Locality: Kotovo Bay (NGR).

Inhabits only Palaearctic waters, where it is widespread and can be attributed even to transpalaearctic group. Predator of small vertebrates and invertebrates. *H. sanguisuga* belongs to very voracious predators, which ingest their prey completely or tear it into big pieces. Our specimens from the south part of transect 1 (Figure 1) were up to 70 mm.

#### 3.1.20. *Erpobdella sp. 1*

 Locality: Kotovo Bay, Sorozhiya Bay, Okunevaya Bay, Cape Kurbulik, Zmejovaya Bay, and Krokhalinaya Bay.

This taxon was recently listed for Lake Baikal by Kaygorodova [17]. *Erpobdella* sp. 1 is widespread in the eutrophic and mesotrophic zones of the Chivyrkuy Gulf (Figure 1: transects 1 and 2). Depending on the environmental conditions this animal could be predator of small invertebrates, necrophage or detritophage. The large-sized leeches are about 50 mm in length and 4-5 mm in width.

#### 3.1.21. *Erpobdella sp. 2*

 Locality: Monakhovo Bay, Sorozhiya Bay, Okunevaya Bay, Cape Kurbulik, and Zmejovaya Bay.

These leeches were registered for the first time in Baikal by Kaygorodova [16]. They were found in the coastal zone of the Chivyrkuy Gulf from Monakhovo Bay to Zmejovaya Bay (Figure 1). Biology of this species is similar to that of other representatives of the genus. Predator of small invertebrates, necrophage or detritophage. Large-sized leeches are up to 40 mm long and 3–5 mm wide.

#### 3.1.22. *Erpobdella sp. 3*

 Locality: Zmejovaya Bay.

For the first time reported in Baikal. These leeches were found only in Zmejovaya Bay (Figure 1: transect 2). Predator of small invertebrates, necrophage or detritophage. Specimens differ from *Erpobdella* sp. 1 and *Erpobdella* sp. 2 by dark dorsal pigmentation.

## 4. Discussion

### 4.1. Species Diversity

At present, 22 species and 2 subspecies from two orders, Rhynchobdellida (18 sp.) and Arhynchobdellida (4 sp.), four families, Glossiphoniidae (6 genera: 13 species and subspecies), Piscicolidae (3 genera: 5 species), Haemopidae (1 genus: 1 species), and Erpobdellidae (1 genus: 3 species), have been revealed in the Chivyrkuy Gulf of Lake Baikal and documented in this paper. This species diversity includes five widespread Holarctic and Palaearctic species—*Hemiclepsis marginata*, *Helobdella stagnalis*, *Glossiphonia complanata*, *Alboglossiphonia heteroclita*, and *Haemopis sanguisuga*; two rare Palaearctic species—*Theromyzon maculosum* and *Helobdella nuda*. In addition, six species belonging to three endemic to Baikal genera (*Baicaloclepsis*, *Baicalobdella*, and* Codonobdella*) were found in the Chivyrkuy Gulf. *Baicaloclepsis echinulata*, *Baicaloclepsis *sp., *Codonobdella truncata*, *Baicalobdella cottidarum*,* Baicalobdella torquata*, and *Baicalobdella* sp. inhabit deep oligotrophic zones (Figure 1: transects 3 and 4), which are near to open waters of the Baikal proper.

Some Palaearctic species of the checklist have been recorded in Eastern Siberia for the first time—*Glossiphonia concolor*, *Alboglossiphonia hyalina*, and both forms of* A*. *heteroclita* f. *papillosa* and f. *striata*. One missing species of our collection is *Theromyzon tessulatum*. Nevertheless, it is involved in the final species list of the Chivyrkuy Gulf leech fauna, so far as its presence in this gulf has never been contradicted and it is highly expected to be found there. At the same time, *Acanthobdella peledina* Grube, 1851, *Piscicola geometra* (Linnaeus, 1761), and *Cystobranchus mammillatus* (Malm, 1863) were excluded from the species list of Lake Baikal. We have never found these three fish parasites in Baikal. It has been proved that Snimschikova [24] erroneously put *A. peledina* into the list of Baikal leeches [27, 28]. We agree that *P. geometra* has wide distribution throughout the entire territory of the former USSR except for waters of Kamchatka and Lake Baikal [18] and that distribution of *C. mammillatus* is confined to northern waters including large tributaries of Lake Baikal such as the Selenga River (found at a distance of 1.5 km from Lake Baikal) and the Upper Angara River, but never Lake Baikal itself [27]. Both piscicolid species have been wrongly listed previously for the lake [12, 24, 29].

Moreover, eight unspecified leech taxa were detected in the Chivyrkuy Gulf, namely, *Glossiphonia *sp., *Baicaloclepsis *sp., *Baicalobdella* sp., two taxa of the genus *Piscicola*, and three taxa of the *Erpobdella*. Most likely, these worms are new to science species awaiting description. Two species *Erpobdella* sp. 1 and *Erpobdella* sp. 2 were presented in the previous studies [16, 17], whereas *Glossiphonia* sp., *Baicaloclepsis* sp., *Baicalobdella *sp., and both *Piscicola* species and *Erpobdella* sp. 3 are new findings and probably endemic species to Lake Baikal. As a result, the Baikal species list of parasite fauna was supplemented with new findings including new species.

The Baikal fauna of parasitic annelids had not been a target of a study for a long time. Information on these parasites appeared often accidentally during benthic or ichthyologic research. Data on the species composition data were so precarious and contradictory that to go on relying on them means to go downwards. For instance, previous researchers declared 8, 18, 13, or 4 leech species in the Baikal ([12], [24], [30], [29], resp.). A recent taxonomic study indicates the existence of 22 species of these parasites in the lake [16, 17]. Taking into account the latest data on the Chivyrkuy Gulf, the species list of the Lake Baikal Hirudinea has been presently expanded up to 30 taxa.

The specificity of annelid parasites lies in difficulty of inventory of their fauna. For example, the undercount of piscine parasites could be explained by their “escape” from moribund animals and by short duration of the parasitic phase in the leech life cycle, as evidenced by their absence in the studied structure of benthic parasites of *Coregonus baicalensis* [31] and *Rutilus lacustris* [32]. Therefore, the species composition of leech fauna of the Chivyrkuy Gulf presented in this paper can be replenished in the future with fish parasites mainly.

### 4.2. Species Distribution

Information about the occurrence of each leech taxa within the Chivyrkuy Gulf is summarized in Table 2. The distribution of leech diversity within the entire gulf space is irregular or nonuniform. Species composition of each area exists as a certain community where many species cooccur and interact. This statement is known as one of the main rules that are universally true in community ecology [33]. Within most natural assemblages a few species comprise the majority of the individuals and are called dominant species. This relationship is termed the distribution-abundance relationship. As follows from Table 2, four species have the most extended ranges within the Chivyrkuy Gulf—*Helobdella stagnalis*, *Glossiphonia complanata*, *Erpobdella* sp. 1, and *Erpobdella* sp. 2. Of them, *Erpobdella* sp. 1 is the most abundant and prevalent species in the Chivyrkuy Gulf. It was found all over the west coast from the Kotovo Bay to the Zmejovaya Bay except the Monakhovo, as well as in the east part of the gulf in the Krokhalinaya Bay. Widespread Palaearctic species *H. stagnalis* is also common in the Chivyrkuy Gulf. It is distributed continuously along the western coast of the gulf occurring in all bays from Kotovo to Zmejovaya (Figure 1, Table 2) but was never found on the rocky east coast.

We noticed that two species *G. complanata* and *Erpobdella* sp. 2 live side by side, often occurring together on the same substrate along the western coast from the Monakhovo Bay to the Zmejovaya Bay. Such coexistence was observed in the aquarium too. Predaceous erpobdellas eat away molluscs weakened by bloodsucking glossiphonia. In the absence of glossiphonia, erpobdellas are unable to cope with molluscs. Molluscs have a chance to survive in the aquarium if kept with a few glossifonia or any number of erpobdellas separately.

The most abundant species of leeches in the Chivyrkuy Gulf hence have two patterns of species distribution. If the pair *Erpobdella* sp. 1 and *H. stagnalis* exists independently of one another under the same ecological conditions, then another pair of dominant species *G. complanata* and *Erpobdella* sp. 2 is a vivid example of a noncompetitive or even mutually advantageous cooccurrence. Both patterns are among the most robust patterns in community ecology [34, 35]. A generalist *Erpobdella* sp. 2 enables *G. complanata* to use benefits of a broad range of environmental conditions, while a food specialist *G. complanata* helps its partner to use a wider range of resources.

Some species of the Chivyrkuy Gulf prefer to live under certain ecological conditions and therefore have a restricted geographic range. These are as follows: *Theromyzon maculosum* (Monakhovo bay) and *Haemopis sanguisuga* (Kotovo bay). *Helobdella nuda* and *Erpobdella* sp. 3 were found exclusively within the Zmejovaya bay, whereas *Hemiclepsis marginata* and *Glossiphonia concolor* live in the Kotovo as well. *Glossiphonia* sp. was found in both Zmejovaya Bay and Monakhovo Bay. Widespread Palaearctic species *T. maculosum* and *H. sanguisuga* are likely to demand certain environmental conditions. *Helobdella nuda* and *Erpobdella* sp. 3 are likely to be scarce species and occur locally in low densities. With regard to *G. concolor* and *Glossiphonia* sp. mentioned in Lake Baikal for the first time, they are also not numerous in the Chivyrkuy Gulf and, as it turned out, have a limited spatial distribution.

Six species were found in the outlet of the Chivyrkuy Gulf. All these species belong to Baikal endemic genera—2 species of the genus *Baicaloclepsis* (Toricinae, Glossiphoniidae), 3 species of the *Baicalobdella* (Piscicolidae), and *Codonobdella truncata*. These species are ecological specialists parasitizing Baikal endemic animals and adapted to cold and high oxygen water of the lake.

Species that are restricted in their geographic distribution tend to be scarce whereas widespread species are likely to occur at high densities [36]. This positive interspecific distribution-abundance relationship is intimately related to the patterns in species abundance. Undoubtedly, there is a positive link between measures of a species' success on a local scale (its density) and on a regional scale (its geographic distribution). Although a larger area is more likely to be able to sustain a higher total number of individuals of a species, it is not clear why the number of individuals in a given area should also increase.

**Figure 2:**
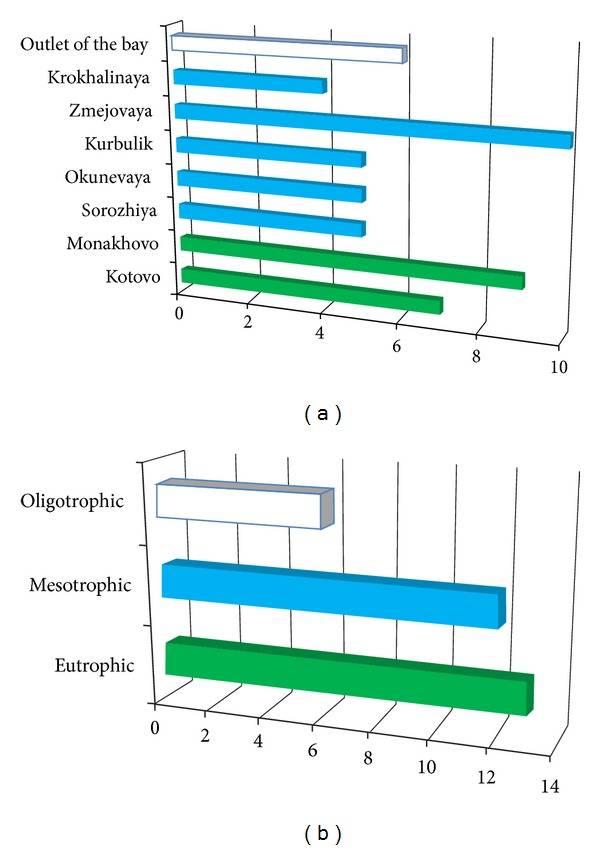
Leech taxa distribution in the Chivyrkuy Gulf. (a) Number of leech species per different bays; (b) number of leech species per each zone with different biological productivity. The abscissa is the number of species and subspecies. Colour of each column corresponds to the water body productivity: eutrophic in green, mesotrophic in blue, and (ultra-)oligotrophic without colour.

The distribution of species also depends on different abiotic and biotic factors; therefore, each bay has its own leech species community (Figure 2). The largest number of leech species inhabits the Zmejovaya Bay (Figure 2(a), Table 2). There were found 10 species belonging to Glossiphoniidae and Erpobdellidae. Restricted inhabitants of this bay are two species *H. nuda* and *Erpobdella* sp. 3 living only there. Krokhalinaya Bay is located on the rocky east side of the gulf. It is populated by the poorest leech community consisting of four species.

We noticed that the leech species diversity is correlated with the bioproductivity status of the bay. It was revealed 14, 11, and 5 leech species were identified in eutrophic, mesotrophic, and oligotrophic zones, respectively (Figure 2(b)). Thus, leech species diversity is reduced from south to north with decrease of eutrophication in the Chivyrkuy Gulf. The exception to this pattern is Zmejovaya Bay, which makes a major contribution to species diversity. Such highest rate of the leech diversity is probably related to the unique natural conditions of this bay. Zmejovaya Bay is rich in hydrothermal springs with temperatures from +39° to +42°C containing sodium sulphates, hydrogen sulphides, and hydrocarbonates [37]. Leech species diversity and abundance which are atypical of mesotrophic zone can be attributed to the effect of specific environmental conditions in the bay. Previously similar impact was found in the Frolikha Bay where an unusually rich community of benthic organisms in the underwater hydrothermal vent influence zone was detected [38].

Most species appear to be limited in at least part of their geographic range by abiotic factors, such as climate, irradiance, temperature, salinity, pH, and so forth. All species have specific limits of tolerance to physical factors that directly affect their survival or reproductive success. Understanding spatial patterns of species diversity and the distributions of individual species is a consuming problem in biogeography and conservation. For hundreds of years biologists have conducted field inventories to map the plant and animal distribution. Yet our understanding of the distribution of most species, especially in remote regions, is still incomplete.

## Figures and Tables

**Table 1:** Leech species composition of the Chivyrkuy Gulf with exact systematic position of all taxa and their taxonomic hierarchy data.

Species	Genus	Subfamily	Family	Suborder	Order	Subclass
*T. maculosum* (Rathke, 1862)	*Theromyzon* Philippi, 1867					
*T. tessulatum*(Müller, 1774)						
*H. marginata *(Müller, 1774)	*Hemiclepsis* Vejdowsky, 1884					
*H. nuda* (Moore, 1924)	*Helobdella* Blanchard, 1876					
*H. stagnalis *(Linn., 1758)						
*G. complanata *(Linn., 1758)	*Glossiphonia* Johnson, 1816					
*G. concolor *(Apáthy, 1888)		**Glossiphoniidae** Autrum, 1936				
*Glossiphonia sp. *						
*A. hyalina *(Müller, 1774)	*Alboglossiphonia* Lukin, 1976					
*A. heteroclita *(Linn., 1761)						
*A. heteroclita *f. *papillosa *(Braun, 1805)			**Glossiphoniidae** Vaillant, 1890			
*A. heteroclita* f. *striata *(Apáthy, 1888)				—	**Rhynchobdellida** Blanchard, 1894	
*B. echinulata *(Grube, 1871)	*Baicaloclepsis* Lukin et Epstein, 1959	**Toricinae** Lukin et Epstein, 1960				**Hirudinea** Lamarck, 1818 (syn. **Hirudinida**)
*Baicaloclepsis *sp.						
*B. cottidarum *Dogiel et Bogolepova, 1957	*Baicalobdella* Dogel et Bogolepova, 1957					
*B. torquata *(Grube, 1871)						
*Baicalobdella* sp.						
*C. truncata *(Grube, 1872)	*Codonobdella *Grube, 1873	**Piscicolinae** Caballero, 1956	**Piscicolidae** Johnston, 1865 (syn. Ichthyobdellidae Leuckart, 1863)			
*Piscicola* sp. 1	*Piscicola *de Blainville, 1818					
*Piscicola* sp. 2						
*H. sanguisuga *(Linn., 1758)	*Haemopis* Sovigny, 1822	—	**Haemopidae** Richardson, 1969	**Hirudiniformes** Coballero, 1952		
*Erpobdella *sp. 1					**Arhynchobdellida** Blanchard, 1894	
*Erpobdella *sp. 2	*Erpobdella *de Blainville, 1818	—	**Erpobdellidae** Blanchard, 1894	**Erpobdelliformes** Sawyer, 1986		
*Erpobdella *sp. 3						

**Table 2:** Geographical distribution of leech species within the Chivyrkuy Gulf of Lake Baikal.

Species/location	Kotovo*	Monakhovo*	Sorozhiya^†^	Okunevaya^†^	Kurbulik^†^	Zmejovaya^†^	Krokhalinaya^†^	Outlet of the gulf^§^
*Theromyzon maculosum *		+						
*Hemiclepsis marginata *	+					+		
*Helobdella nuda *						+		
*Helobdella stagnalis *	+	+	+	+	+	+		
*Glossiphonia complanata *		+	+	+	+	+	+	
*Glossiphonia concolor *	+					+		
*Glossiphonia* sp.		+				+		
*Alboglossiphonia hyalina *		+			+	+	+	
*A. heteroclita * f. *papillosa *	+	+		+			+	
*A. heteroclita* f. *striata *	+	+						
*Baicaloclepsis echinulata *								+
*Baicaloclepsis *sp.								+
*Baicalobdella cottidarum *								+
*Baicalobdella torquata *								+
*Baicalobdella* sp.								+
*Codonobdella truncata *								+
*Piscicola* sp. 1			+					
*Piscicola* sp. 2		+						
*Haemopis sanguisuga *	+							
*Erpobdella *sp. 1	+		+	+	+	+	+	
*Erpobdella *sp. 2		+	+	+	+	+		
*Erpobdella *sp. 3						+		

Total number of taxa in bay/in zone	7/13	9/13	5/12	5/12	5/12	10/12	4/12	6/6

Symbols displayed in each column correspond to the water body productivity: *eutrophic (green colour in Figure 2), ^†^mesotrophic (blue colour in Figure 2), and ^§^oligotrophic (withot colour in Figure 2).

## Abbreviations

NHR: New host record
NGR: New geographical record
NHPR: New host-parasite record.
